# Data of embedded humidity sensors, sample weights, and measured pore volume distribution for eight screed types

**DOI:** 10.1016/j.dib.2018.09.020

**Published:** 2018-09-12

**Authors:** Christoph Strangfeld, Carsten Prinz, Felix Hase, Sabine Kruschwitz

**Affiliations:** a Bundesanstalt für Materialforschung und -prüfung, Unter den Eichen 87, 12205 Berlin, Germany; b Technische Universität Berlin, Gustav-Meyer-Allee 25, 13355 Berlin, Germany

## Abstract

Four cement-based and four calcium-sulphate-based screed types are investigated. The samples have a diameter of 300 mm and a height of 35 or 70 mm. Up to ten humidity sensors are embedded directly during the concreting of the screed samples. Thus, the humidity over the sample height is monitored during hardening, hydration, evaporation, and oven drying. Furthermore, the screed samples are weighed during every measurement to determine the total mass and the corresponding moisture loss.

To define the pore system precisely, mercury intrusion porosimetry as well as gas adsorption is performed. According to the data, the entire pore volume distribution is known. The measured pore diameters range from 0.8 nm to 100 µm and the total porosity of the examined screeds ranges between 11% and 22%.

Based on these measurement data, moisture transport, pore saturation as well as sorption isotherms and their hysteresis may be calculated quantitatively as described in “Monitoring of the absolute water content in porous materials based on embedded humidity sensors” (Strangfeld and Kruschwitz, 1921).

**Specifications table**

**Table:** 

Subject area	Materials science
More specific subject area	Non-destructive testing in civil engineering
Type of data	Figures
How data was acquired	Capacitive humidity sensors, mercury intrusion porosimetry (MIP), gas sorption, high precision balance
Data format	raw and processed
Experimental factors	Output voltage of the capacitive humidity sensors is converted to relative humidity based on the equation described in the data sheet, pressure steps of MIP are converted to pore radii based on the Washburn equation, pressure steps of gas sorption are converted to pore radii based on the BJH theory
Experimental features	Humidity sensors are embedded directly into screed to monitor material humidity and material moisture
Data source location	Climate chamber at Bundesanstalt für Materialforschung und -prüfung, Unter den Eichen 87, 12205 Berlin, Germany
Data accessibility	Data is available with this article and at http://researchdata.4tu.nl/home/
	DOI: 10.4121/uuid:d2ba436f-78c0–4105-8a1f-5422fcb37851
	URL: https://data.4tu.nl/repository/uuid:d2ba436f-78c0–4105-8a1f-5422fcb37851
Related research article	Strangfeld, C. and Kruschwitz, S, “Monitoring of the absolute water content in porous materials based on embedded humidity sensors”, Construction and Building Materials, vol. 177, pp. 511–521, 2018 [7]

**Value of the data**

• These experimental data might be used to calibrate and validate simulations of moisture transport in porous materials.
• These experimental data of moisture transport in porous materials, including the pore volume distribution, might be used to evaluate and optimize other sorption models.
• These data provide an approach to predict the hysteresis of the sorption isotherm between adsorption and desorption. Other pore geometries and distributions, including tortuosity, might be investigated to describe the hysteresis more precisely.
• These data might help to better understand the shift from convective dominated moisture transport in the pore system at high humidity toward diffusive dominated moisture transport at lower humidity. A linkage of the Navier-Stokes equation to Fick׳s law would be required.
• The proposed data evaluation might be used to adapt the described theory to other porous materials in physics, chemistry, and engineering.

## 1 Data

All examined screed samples are listed in the overview (pdf-file). Eight different screed types were investigated, including four cement-based and four calcium-sulphate-based screeds. Each screed type consisted of three test samples. The first two samples of each screed type were equipped with embedded sensors, the third one was investigated destructively. The weight of the screed samples over time is documented. The corresponding data of the embedded humidity and temperature sensors are given. Furthermore, the data of the pore volume distribution of each screed type based on gas sorption and MIP is documented.

**Fig. 1:**
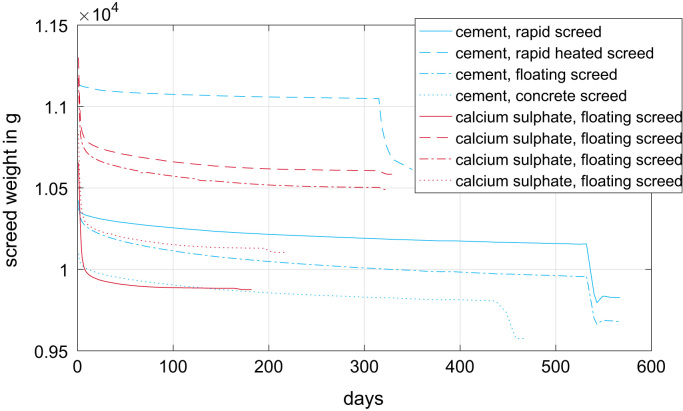
Temporal evolution of the sample weight of the 70 mm high sample of the eight screed types.

Fig. 1 depicts the temporal evolution of the sample weight during hydration and evaporation. In this plot, only the results of the 70 mm high samples of the eight screed types are shown. The same data are available for the 35 mm high samples. The sudden drop in weight, approximately 2 weeks before every measurement stops, is caused by the oven drying. The cement based screeds were dried at 105° C oven temperature, the calcium-sulphate based screeds at 40 °C. Due to the higher drying temperature, the weight drop of the cement based screeds was significantly higher.

**Fig. 2:**
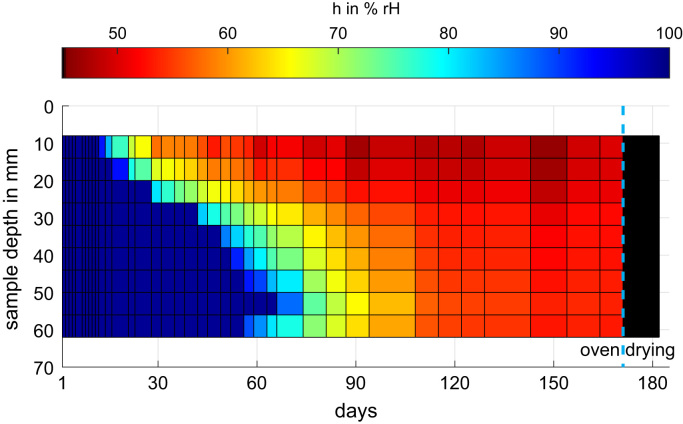
Temporal evolution of the corresponding relative humidity along the sample height based on ten embedded humidity sensors for a calcium-sulphate based floating screed (70 mm high).

Fig. 2 shows the evolution of the corresponding relative humidity for one representative screed sample. The considered screed sample is a 70 mm high calcium-sulphate based floating screed. In the 70 mm high samples, 10 relative humidity sensors were embedded. Every horizontal line represents one measurement position, starting at approximately 8 mm from the top down to 62 mm depth. Every vertical line represents a measurement day- in this case in total 42 measurement days are shown. During the first days, all relative humidity sensors were in saturation state. On day 171, the oven drying started. The corresponding relative humidity decreased below 10% rH. The data of the embedded relative humidity sensors are available for all eight screed types and for the 35 mm and 70 mm high sample as well. Five sensors were embedded in the 35 mm high samples.

**Fig. 3:**
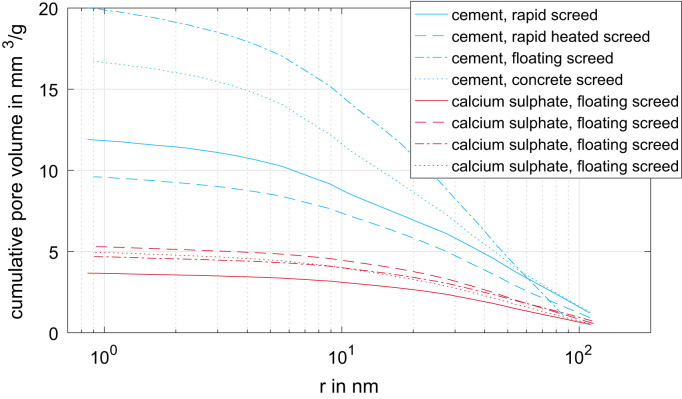
Measured cumulated pore volume distribution of the eight screed types based on gas adsorption.

**Fig. 4:**
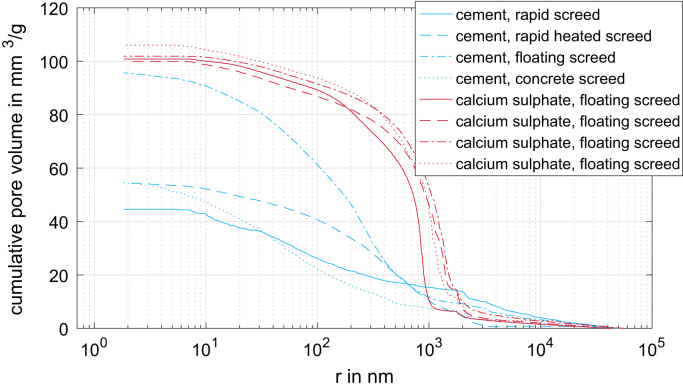
Measured cumulated pore volume distribution of the eight screed types based on mercury intrusion.

Fig. 3 presents the measured cumulated pore volume distribution of the eight screed types based on gas adsorption [2], [6]. The corresponding pore radii range from 0.8 nm up to 100 nm. Similar data are shown in Fig. 4. The measured cumulated pore size distribution is based on mercury intrusion [5], [8]. The corresponding pore radii range from 2 nm up to approximately 50.000 nm.

## 2 Experimental design, materials and methods

Each sample label starts with a D and is followed by a distinct sample number. This sample number is the reference for all measurement data. The sample weight during screed hardening, hydration, water evaporation and oven drying is given in txt-files in the folder “sample weight.” A high precision balance with a maximum load of 72 kg and an accuracy of 0.1 g was used. The file name represents the date of measurement. During some measurement days, not all samples were measured. The third sample of each screed type, e.g. D-03, was investigated destructively. Thus, significant sample weight deviations occurred due to extraction of sample material. From these samples, material for porosity measurements was extracted as well. Two methods were used to investigate the pore volume distribution. The mercury intrusion porosimetry (MIP) applied test pressures between 0.01 MPa and 400 MPa [5]. The conversion from pressure to a certain pore diameter included the Washburn equation [8]. The measurement procedure is the exact reproduction of the international standard [5]. The calculated pore diameter started at approximately 100 µm and the sensitivity was recorded up to a minimum pore diameter of approximately 4 nm. The measurement data are given in a csv-format in the folder “MIP mercury intrusion porosimetry.” In the csv-file, the relevant data are listed in a table called “experimental data.” Column E as “pore size” and column F as “specific pore volume” (cumulated) were used for the prediction of the pore saturation [7]. The other method used was gas adsorption [2], [6] based of the physisorption of nitrogen gas at 77 K in a pressure ranges of 4.5 mbar to 1 bar. The conversion from pressure to a certain pore diameter was based on the BJH theory [1]. The BJH theory includes the layer thickness of the adsorbed nitrogen according to Halsey [3] and the Kelvin equation for calculating the pore radius. The measurement procedure is the exact reproduction of the international standard [6]. The measured pore radii ranged between 0.8 nm and 100 nm. The measurement data of interest are listed in the rpt-data files in the folder “BET gas adsorption.” In the rpt-file, the table of interest has the title “BJH Adsorption Pore Distribution Report.” The second and third column titled “average diameter” and “incremental pore volume” are relevant for moisture calculations [7]. Although MIP is more appropriate for larger pore and gas adsorption for micropores, both methods overlap each other in the mesopore range of approximately 2 nm to 100 nm. Thus, a sensible combination of the two porosimetry results has to be found for further analysis regarding the moisture calculations [7].

The humidity and temperature data are saved in the folder “embedded sensors.” Only non-destructively tested samples were equipped with embedded sensors. The temperature sensors were MCP9700A three-pin thermistors and the humidity sensors were HIH-5031 sensors [4]. All sensors were in surface mounted device design. The samples of 35 mm height contained 5 sensors, the samples of 70 mm height contained 10 sensors. For each sample, one txt-file with all sensor data exists. The first column shows the date, the second the daytime. Then, the temperature data and the corresponding standard deviation of each sensor are listed. The humidity data are given in percent. The first column with humidity data represents the topmost sensor in the sample. Every humidity value stands for a mean value of 160 single measurements recorded with 16 Hz over 10 s. The standard deviation of these measurements is given in the data file as well. The last columns represent measurements of the electrical resistivity and electrical capacity based on the measurement of embedded multi-ring electrodes. If the measured temperature increased to 40 °C or 105 °C, the samples were placed in the drying oven to get the final dry mass.
